# In dogs with chronic enteropathy (idiopathic inflammatory bowel disease) is budesonide more effective than prednisolone or prednisone in resolving clinical signs?

**DOI:** 10.18849/ve.v8i4.673

**Published:** 2023-12-13

**Authors:** Victoria Alice Coates+

**Keywords:** BUDESONIDE, CANINE, CIBDAI, DOGS, ENTEROPATHY, INFLAMMATORY BOWEL DISEASE, IBD, PREDNISOLONE, PREDNISONE

## Abstract

**PICO question:**

In dogs with chronic enteropathy (idiopathic inflammatory bowel disease) is budesonide more effective than prednisolone or prednisone in resolving clinical signs or improving the canine inflammatory bowel disease (IBD) activity index (CIBDAI) or the canine chronic enteropathy clinical activity index (CCECAI)?

**Clinical bottom line:**

**Category of research:**

Treatment.

**Number and type of study designs reviewed:**

One double-blinded randomised control trial.

**Strength of evidence:**

Moderate.

**Outcomes reported:**

The outcome of this study identified that no significant differences in remission rates (> 75% decrease in CIBDAI scores) were observed between the prednisone and budesonide groups. Frequency of adverse effects were also similar between the two groups.

**Conclusion:**

A single study with moderate power of evidence alongside some significant limitations, particularly population size, cannot be used as the sole provider of an answer to the PICO question. As such, further studies with greater power would be required before a definitive assessment of comparative treatment efficacy can be made.

## How to apply this evidence in practice

The application of evidence into practice should take into account multiple factors, not limited to: individual clinical expertise, patient’s circumstances and owners’ values, country, location or clinic where you work, the individual case in front of you, the availability of therapies and resources.

Knowledge Summaries are a resource to help reinforce or inform decision making. They do not override the responsibility or judgement of the practitioner to do what is best for the animal in their care.

## Clinical scenario

A veterinarian working in clinical practice has diagnosed a 5-year-old female neutered cross breed dog with a chronic enteropathy (idiopathic inflammatory bowel disease) through a lack of response to dietary and microbiome manipulations, and identification of lymphoplasmacytic inflammation on mucosal biopsies obtained via gastroduodenoscopy. The veterinarian would like to start the dog on the most effective first-line immunosuppressive medication to improve the dog’s Canine Inflammatory Bowel Disease Activity Index (CIBDAI) score and is wondering whether budesonide would be more effective than prednisone.

## The evidence

The search identified one double-blinded randomised treatment control trial that was related to the PICO question. The experimental design enabled the provision of a high strength of evidence as the study was well planned, patient follow-up was good, and the study was conducted in a clinical setting. The main limitation of the study was the small sample size limiting the power of the statistical analysis.

## Summary of the evidence

**Dye et al. (2013):** 

Population:	Dogs more than 3 kg in bodyweight with a diagnosis of idiopathic inflammatory bowel disease (IBD).
Sample size:	40 dogs.
Intervention details:	Diagnostic interventions before entry into the study: Complete blood count and biochemistry profile. Urinalysis. Abdominal ultrasound. Faecal direct smear and zinc sulphate flotation. Histopathological review of mucosal biopsy specimens (obtained from gastroduodenoscopy, colonoscopy or both) performed by a single board-certified pathologist. Treatment regimen: Dogs were randomised by a computer-generated schedule into one of two treatment groups. The prednisone group (n = 20) received prednisone per os at a dosage of 1 mg/kg q12h for 3 weeks, then 0.5 mg/kg q12h for 3 weeks. The budesonide group (n = 20) received powder-based budesonide in capsule form with dosage based on bodyweight as follows: 3–7 kg: 1 mg budesonide q24h; 7.1–15 kg: 2 mg budesonide q24h; 15.1–30 kg: 3 mg budesonide q24h; > 30 kg: 5 mg budesonide q24h. The dosage was continued for the 6-week duration of the study. No other medications were administered for treatment of idiopathic IBD during the 6-week study period. Antibiotics were administered for other conditions (urinary tract infection, pyoderma) if indicated. Dogs were continued on the same diet; dietary changes were not allowed during the study period. Evaluations: Owners were contacted on a weekly basis to complete a verbal questionnaire regarding their pet’s clinical signs and attitude. Dogs were examined by one of the study authors at 3 weeks and 6 weeks after initiation of treatment. Complete blood count, biochemistry profile and urinalysis were repeated at 3 weeks and 6 weeks. Gastrointestinal endoscopy and biopsies with histopathology were repeated at 6 weeks.
Study design:	Double-blinded randomised control treatment trial.
Outcome studied:	Outcomes studied: Information from the client questionnaire and medical record regarding the dog’s attitude, appetite, vomiting, faecal consistency, and faecal frequency was used to calculate the CIBDAI at enrolment and at 6 weeks. Scores were calculated by a study author who was not involved in case management and was blinded to the dog’s treatment group. Clinical remission was defined as a 75% reduction in the CIBDAI score from the pretreatment value.
Main Findings (relevant to PICO question)	Four dogs in the prednisone group did not complete the study. Both treatments were effective in treatment of IBD with significant (P = < .0001) reduction in CIBDAI scores and similar overall remission rates.
Limitations:	Study population was small, limiting the power of the statistical analysis. Patient management and trial therapies were not standardised before entering the study. Medical records prior to referral were often incomplete, thus making it difficult to assess the adequacy of the trials. Study was limited to a 6-week duration. Large number of dogs with eosinophilic inflammation in the prednisone group, which could have affected response to the treatment. Dogs in the prednisone group had higher white blood cell counts and neutrophil counts, which could indicate greater disease severity in this population of dogs.

## Appraisal, application and reflection

The literature search revealed one paper that was directly relevant to the PICO question outlined; this was a randomised double-blinded treatment control study (Dye et al., 2013). This study design method provides a high strength of evidence when assessing treatment efficacy due to the minimisation of bias. The study compared a population of dogs diagnosed with idiopathic inflammatory bowel disease (IBD) treated blindly with either budesonide or prednisone. Budesonide, a non-halogenated glucocorticoid, was developed to limit systemic side effects in human patients with IBD; whereas prednisone is a glucocorticoid prodrug metabolised by the liver to its active metabolite prednisolone, which results in systemic side effects when administered (Becker, 2013). The diagnostic procedures before entry into and upon completion of the trial, alongside treatment protocols, were standardised. Evaluations conducted throughout the study included weekly owner questionnaires regarding their dog’s clinical signs and attitude, alongside repeat physical examination by one of the study authors 3 weeks and 6 weeks after the initiation of treatment. Information gathered enabled the calculation of CIBDAI score both at enrolment and at 6 weeks, with clinical remission being defined as a 75% reduction in the pretreatment CIBDAI score. The study found that there was no significant difference (P = < .0001) in the effectiveness of budesonide compared to prednisone in improving the CIBDAI scores of the study population; both treatments had similar overall remission rates.

Despite providing statistically significant results, this study had multiple significant limitations that contributed to the weakness of the evidence. Firstly, the small size of the study population limited the power of the statistical analysis. Alongside this, management and trial therapies were non-standardised before entering the study, and medical records prior to referral were often incomplete; thus, assessing the true adequacy of the trial is difficult. Although randomised, the groups were not well matched at baseline as the dogs receiving prednisone therapy both had a greater proportion of eosinophilic inflammation, alongside higher white blood cell and neutrophil counts. Both these differences could have impacted on the study results; firstly, through the effect eosinophilic inflammation could have on response to treatment, and secondly through the indication of a greater disease severity in dogs in the prednisone group. Finally, the study was limited to a 6-week period; this unfortunately limits the findings of this study to be not representative of the long-term use of these immunomodulatory medications.

Based on the strength of the study analysed, there is only weak evidence comparing the efficacy of prednisone and budesonide when treating dogs with idiopathic IBD. Despite this, the non-inferiority of budesonide in comparison to prednisone is encouraging, particularly as this has previously been reported to have reduced systemic side effects observed in dogs (Pietra et al., 2013; Tumulty et al., 2004; and Stroup et al., 2006). However, our analysed study by Dye et al. (2013) is underpowered and did not identify any significant difference in observed side effects between budesonide and prednisone, indicating further research is needed in these areas.

Overall, a single study with moderate power of evidence of alongside some significant limitations, particularly population size, cannot be used as the sole provider of an answer to our PICO question. As such, further studies with greater power would be required before a definitive assessment of comparative treatment efficacy in reducing CIBDAI scores can be made.

## Methodology

**Search strategy:** 

Databases searched and dates covered:	CAB Abstracts on the OVID interface (1973 to 28/04/2023) PubMed on the NCBI interface (1920 to 28/04/2023)
Search strategy:	CAB Abstracts: (dog or dogs or canine or canines or bitch or bitches).mp. or exp dogs/ or exp bitches/ (inflammatory bowel disease or IBD or inflammatory enteropath* or chronic enteropath*).mp. or exp inflammatory bowel diseases/ (budesonide or Pulmicort or Budenofalk or Entocort).mp. (prednisolone or prednisone or prednicare or prednidale or dermipred or deltasone or sterapred or prednis-tab).mp. or exp prednisolone/ or exp prednisone/ 1 and 2 and (3 or 4) PubMed: #1 dog or canine or bitch #2 inflammatory bowel disease or IBD or inflammatory enteropathy or chronic enteropathy #3 budesonide or Pulicort or Budenfalk or Entocort #4 prednisolone or prednisone or prednicare or prednidale or dermipred or deltason or sterapred or predis-tab #5 #1 and #2 and (#3 or #4)
Dates searches performed:	28 Apr 2023

**Exclusion / inclusion criteria:** 

Exclusion:	Non-English language, popular press articles, human literature.
Inclusion:	Any relevant primary veterinary research or systematic review which examined the efficacy of budesonide and prednisolone in resolving the clinical signs or improving the canine IBD activity index (CIBDAI) or the canine CE clinical activity index (CCECAI).

**Search outcome:** 

Database	Number of results	Excluded – Did not answer the PICO question	Excluded – Not English language	Excluded – Conference abstract only	Excluded – Human literature	Excluded – Duplicates	Total relevant papers
CAB Abstracts	64	60	0	3	0	0	1
PubMed	62	56	0	0	5	0	1
Total relevant papers when duplicates removed							1

## ORCiD

Victoria Alice Coates: https://orcid.org/0009-0006-5322-5387

## Conflict of interest

The author declares no conflicts of interest.

## Disclaimer

Knowledge Summaries are a peer-reviewed article type which aims to answer a clinical question based on the best available current evidence. It does not override the responsibility of the practitioner. Informed decisions should be made by considering such factors as individual clinical expertise and judgement along with patient’s circumstances and owners’ values. Knowledge Summaries are a resource to help inform and any opinions expressed within the Knowledge Summaries are the author’s own and do not necessarily reflect the view of the RCVS Knowledge. Authors are responsible for the accuracy of the content. While the Editor and Publisher believe that all content herein are in accord with current recommendations and practice at the time of publication, they accept no legal responsibility for any errors or omissions, and make no warranty, express or implied, with respect to material contained within. For further information please refer to our Terms of Use.
